# One-Step Chemical Vapor Deposition Synthesis of 3D N-doped Carbon Nanotube/N-doped Graphene Hybrid Material on Nickel Foam

**DOI:** 10.3390/nano8090700

**Published:** 2018-09-07

**Authors:** Hua-Fei Li, Fan Wu, Chen Wang, Pei-Xin Zhang, Hai-Yan Hu, Ning Xie, Ming Pan, Zheling Zeng, Shuguang Deng, Marvin H. Wu, K. Vinodgopal, Gui-Ping Dai

**Affiliations:** 1 Institute for Advanced Study, Nanchang University, Nanchang 330031, China; hfli@email.ncu.edu.cn (H.-F.L.); nxie@email.ncu.edu.cn (N.X.); 2 School of Resources Environmental & Chemical Engineering, Nanchang University, Nanchang 330031, China; fwu@email.ncu.edu.cn (F.W.); c.wang@email.ncu.edu.cn (C.W.); peixin.zhang@email.ncu.edu.cn (P.-X.Z.); hyhu@email.ncu.edu.cn (H.-Y.H.); mpan@email.ncu.edu.cn (M.P.); zlzengjx@ncu.edu.cn (Z.Z.); shuguang.deng@asu.edu (S.D.); 3 Key Laboratory of Poyang Lake Environment and Resource Utilization, Nanchang University, Ministry of Education, Nanchang 330031, China; 4 School for Engineering of Matter, Transport and Energy, Arizona State University, Tempe, AZ 85287, USA; 5 Department of Physics, North Carolina Central University, Durham, NC 27707, USA; mwu@nccu.edu; 6 Department of Chemistry and Biochemistry, North Carolina Central University, Durham, NC 27707, USA

**Keywords:** N-doped CNTs, N-doped graphene, 3D hybrid, melamine, CVD synthesis

## Abstract

3D hybrid nanostructures connecting 1D carbon nanotubes (CNTs) with 2D graphene have attracted more and more attentions due to their excellent chemical, physical and electrical properties. In this study, we firstly report a novel and facile one-step process using template-directed chemical vapor deposition (CVD) to fabricate highly nitrogen doped three-dimensional (3D) N-doped carbon nanotubes/N-doped graphene architecture (N-CNTs/N-graphene). We used nickel foam as substrate, melamine as a single source for both carbon and nitrogen, respectively. The morphology and microstructure were characterized by scanning electron microscopy, transmission electron microscopy, X-ray diffraction, isothermal analyses, X-ray photoelectron microscopy and Raman spectra. The obtained 3D N-CNTs/N-graphene exhibits high graphitization, a regular 3D structure and excellent nitrogen doping and good mesoporosity.

## 1. Introduction

A viable approach to achieving a robust 3D hybrid architecture is by integrating different low-dimensional nanostructures [1]. Graphene, a hexagonal 2D nanostructure composed of regular sp^2^ hybridized carbon atoms, shows outstanding electrical conductivity and mechanical strength [2,3,4,5,6,7,8,9,10,11]. Carbon nanotubes (CNTs) are typical one-dimensional nanomaterials with excellent performance. Owing to the unique mechanical strength, large surface-to-volume ratio and high electrical property, CNTs have become a promising choice for potential applications, such as energy storage [12], supercapacitors, batteries [13] and nanoelectronic devices [14]. Nevertheless, in the synthesis process of graphene and CNTs, there is a tendency for irreversible aggregation and stacking due to van Waals interactions [10,14]. Consequently, the attainable properties decline compared to theoretical predictions. The synthesis of three-dimensional carbon architectures from CNT and graphene effectively reduces the aggregation and stacking which occur among layers of graphene and CNTs [15]. 3D carbon architectures built from CNTs and graphene has attracted numerous attention [16,17,18,19] due to the extraordinary mechanical and electrical properties and potential possibilities in a variety of applications for example, lithium-sulfur batteries [20], supercapacitors [21] and energy storage [22].

To improve the electrical and chemical properties, various strategies have been attempted by scientists. One of the effective approaches is introducing heteroatom doping, for example, boron, sulfur, phosphorus and nitrogen [23,24,25]. Nitrogen, has smaller atomic radius and higher electronegativity than carbon and hence is a desirable dopant to improve electronic properties and surface wettability [7]. The nitrogen-doping could induce more defaces and active sites for improve interfacial adsorption and effectively trap lithium polysulfides at electroactive sites within the cathode [26]. However, N-doped carbon nanostructure reported in recent years mainly includes typical chemical vapor deposition (CVD), graphene treated with nitrogen plasma, thermal annealing graphene oxide (GO) under NH_3_ atmosphere and approaches based on different templates. Among them, CVD is the wide method for the synthesis of N-doped carbon nanotube and graphene, which use flammable organic gases (e.g., CH_4_, C_2_H_4_) or toxic organic solvent (e.g., benzene) and pyridine or NH_3_ as carbon source and nitrogen source, respectively. And the nitrogen N-doped hybrids are always fabricated by a multistep CVD route [20,27,28,29,30,31]. Zhu et al. reported the two-step CVD approach, which using CH_4_ and C_2_H_2_ as carbon source to synthesize carbon nanotubes/graphene hybrid [27]. Moreover, thermally and functional toxic catalysts are necessary for growth of hybrid nanostructure. Yan et al. fabricate desirable three-dimensional N-doped mesoscopic carbon material under combined function of Fe-Co-Ni catalysts [28]. Dong et al. and Su et al. synthesized the N-doped carbon nano-architecture by utilizing different precursors which need more procedures [20,29]. Wang et al. and Ding et al. used CVD method to synthesize the N-doped carbon nanotubes/graphene structure, where the (graphene oxide) GO and glucose are used as carbon source, respectively and the melamine only used as nitrogen source [7,30]. Samad et al. have employed polyurethane (PU) as a source of N-doping graphene foam by using a two-step technique [31]. Therefore, design and synthesis of 3D high N-doped carbon nanotubes /N-doped graphene by using a facile and reasonable low-cost strategy is still a significant challenge.

In this study, we reported, for the first time, one-step CVD method to synthesize three-dimensional nanostructure consisting of N-doped carbon nanotubes/N-doped graphene (N-CNTs/N-graphene) can be grown on a nickel foam substrate using low-cost material melamine as a single source for both carbon and nitrogen, respectively. In this contribution, nickel foam, a three-dimensional interconnected structure, not only acts as a porous 3D scaffold for generating graphene and carbon nanotube but also simultaneously provides situ generated Ni nanoparticles (Ni NPs) which facilitate the nucleation and growth of N-doped carbon nanotubes (N-CNTs) on the surface of the N-doped graphene (N-graphene) without requiring any other synthetical catalyst. Our process enables the synergistic use of hydrogen to remove the impurities on the surface of nickel foam, boost the growth of graphene and carbon nanotubes by etching layered carbon nitride which was produced from melamine pyrolysis.

## 2. Materials and Methods

### 2.1. Synthetic Procedures of N-CNTs/N-Graphene

**Figure 1:**
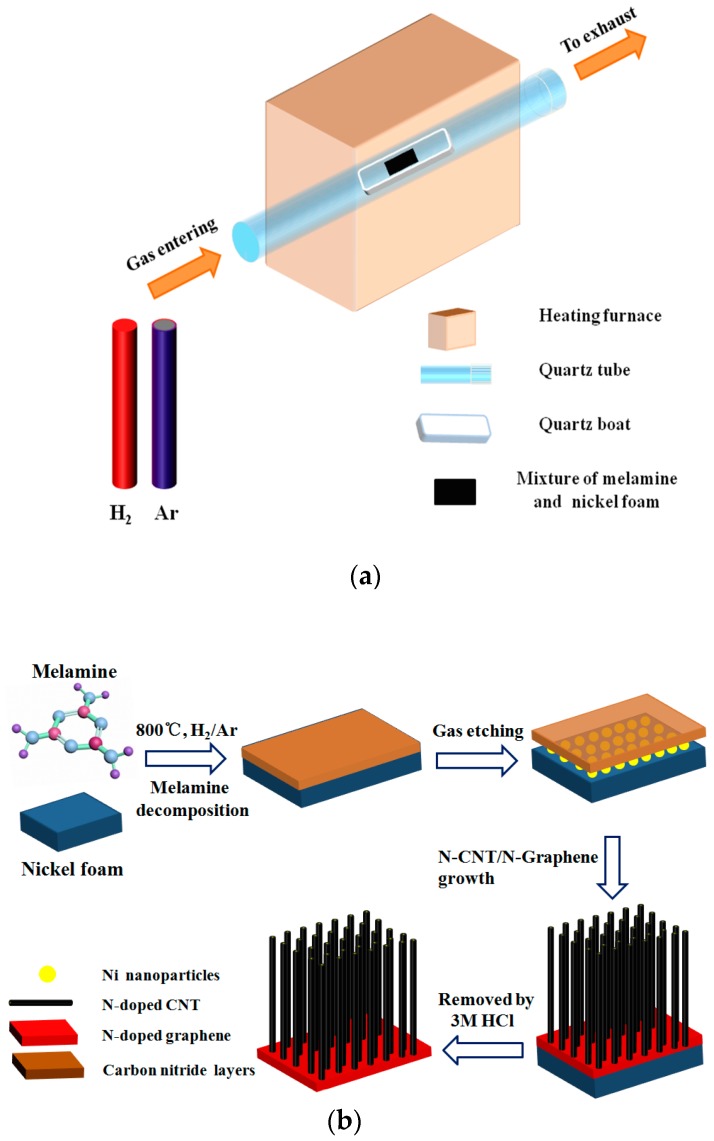
(**a**) Illustration of the main synthetic device of N-CNTs/N-graphene hybrid; (**b**) Schematic diagram of the of N-CNTs/N-graphene material synthesized by one-step CVD method.

Nickel foam (NF) were first cut into pieces of 10 × 10 mm^2^ and successively dispersed in acetic acid solution and ethanol solution by ultrasound for 10 min to clean their surfaces and remove the thin surface oxide layer. Subsequently, the NF was dried under nitrogen (99.99%) atmosphere. The NF thus obtained and melamine was mixed according to the mass ratio (1:5) and the above mixture was then placed in a horizontal quartz tube with outer diameter of 30 mm and inner diameter of 22 mm. Before the CVD reactor (Figure 1a) was heated to 600 °C, H_2_ (99.99%) was introduced for about 20 min at a flow of 70 sccm. When the center of the furnace reached a temperature of about 800 °C, the sample was annealed for 0.5 h at this temperature under a mixed gas (the flow rate of Ar and H_2_ is 5:1) atmosphere. After that, the mixture was cooled to room temperature in Ar atmosphere at a flow of 30 sccm and the products were taken out of the quartz tube. Finally, the black product was placed in a 3M HCl solution at 80 °C for two days to fully remove the nickel foam yielding the desired 3D N-CNTs/N-graphene. Furthermore, to obtain uniform N-CNTs growth in this study, we carried out experiments by using different mass ratio between NF and melamine (1:1, 1:5 and 1:10) as shown in Figure S1.

### 2.2. Characterization

The elemental analysis and crystalline degree of the prepared products was characterized with X-ray diffraction (XRD, Bruker D8 Advance, Billerica, MA, USA). Field-emission scanning electron microscopy (SEM, FEI QUANTA 200F, Hitachi, Tokyo, Japan) and transmission electron microscopy (TEM, JEOL 2010F, Peabody, MA, USA) was used to observe the morphologies and whole structure of the product. The surface-to-volume ratio of material and pore structures were measured using nitrogen adsorption and desorption isotherms by a Quanta Chrome adsorption instrument (ASAP 2460, Micromeritics, Norcross, GA, USA). X-ray photoelectron spectroscopy analyses (XPS, PHI-5700, Ulvac-Phi, Chigasaki, Japan) and Raman spectroscopy (Raman, Horiba Evolution, Tokyo, Japan) was used to analyze the product.

## 3. Result and Discussion

The whole synthetic process of 3D N-CNTs/N-graphene involves one-step CVD in the solid-state pyrolysis of melamine at 800 °C as shown in Figure 1a. The possible growth mechanism during heating process is outlined as follows (Figure 1b). First, at a temperature below 300 °C, melamine was absorbed and uniformly distributed on the surface of NF. Melamine has a bi-functional effect in this work a) providing carbon nitride for the growth of carbon nanotube and graphene and b) creating Ni nanoparticles (Ni NPs) which appeared due to etching process of NH_3_ [7]. Between 300 °C to 600 °C, melamine gradually decomposed to carbon nitride and released NH_3_ which enables the growth of CNTs [32]. There are many no-uniform amorphous carbon and pores distribute on the NF in Figure S2, indicating the decomposition of melamine and gases are released from the pores. With the increasing temperature, layered graphitic carbon nitride deposits on the surface of NF and gradually decomposes to graphene under the effective etching process of H_2_ about 800 °C. Simultaneously, carbon nanotube grows on the surface of graphene layers catalyzed by the Ni nanoparticles produced by etching process of NH_3_ resulting from decomposing of melamine. In addition, it can be observed that the growth process of CNTs is based on a “tip growth” mechanism [33] shown below. The growth temperature of graphene is largely related to the species of carbon source. In this work, the melamine, solid carbon and nitrogen source, is used as a feedstock. Compare to most of gaseous carbon sources, melamine decomposes at a lower temperature [32]. Therefore, solid carbon resource may be a better resource due to the fast carbon diffusivity through Ni foam and coating on the surface at low temperature. Especially during the dehydrogenation process of gaseous carbon sources, a higher temperature is always required to populate high energy intermediates and thus, the overall effective dehydrogenation barrier and nucleation barrier of gaseous carbon resources are much higher than that of solid carbon sources [9]. The one-step growth in this work provides a facile and relative low-temperature way for synthesis of 3D N-doped hybrids compared to other CVD routes, which is consistent with the related reports which fabricate graphene by using solid as carbon source [9].

**Figure 2:**
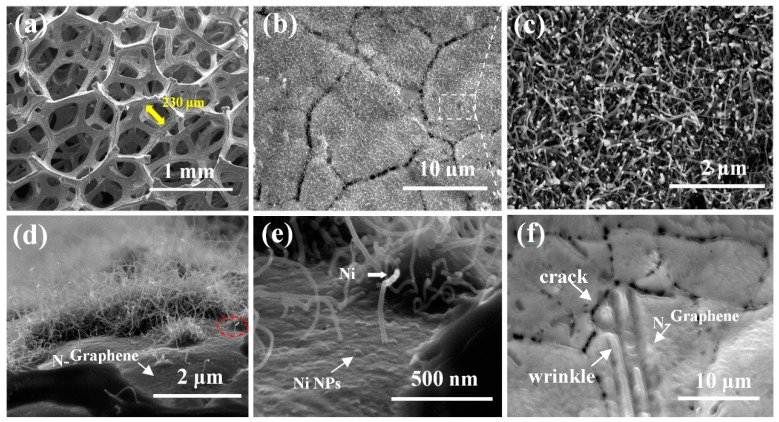
(**a**) SEM image of the N-CNTs/N-graphene hybrid grown on the surface of nickel foam; (**b**,**c**) The top view of low and high magnification SEM images of the N-CNTs; (**d**,**e**) Low-magnification and high-magnification SEM images of one side view of N-CNTs and N-graphene on the surface of the nickel foam; (**f**) SEM image of the only N-graphene layers grown on the surface of nickel foam.

The structure and morphology of N-CNTs/N-graphene can be observed by field-emission scanning electron microscopy (SEM). Figure 2a shows the porous 3D interconnected structure of NF and the N-CNTs/N-graphene synthesized after high-temperature reaction. Some cracks can be obviously observed on the surface of NF as displayed in Figure 2b,f, which could be due to the difference in thermal expansion coefficients of nickel foam during heating process [34]. Moreover, Figure 2c displays the uniformly and densely distributed N-CNTs growing on the surface of NF covered by N-graphene. For the sake of specificity, the experiment condition to be changed is mass ratio of NF and melamine while the other external conditions were maintained constant. The SEM images were obtained to determine the morphology of the N-CNTs, as shown in Figure S1. There is a change in the surface morphology when the mass ratio of NF and melamine increases from 1:1 to 1:5. N-CNTs are seen in the samples grown at mass ratio of 1:1 (Figure S1a) and have sparse and uneven distribution on the surface of graphene. When the mass ratio is increased to 1:5, uniform and denser N-CNTs were obtained as shown in Figure S1b. However, Figure S1c reveals that increasing the mass ratio (1:10) of NF and melamine leads to the formation of non-uniform CNTs with different diameters. These results as a whole suggest that the growth and surface coverage density of CNTs is sensitive to mass ratio with an optimal ratio of 1:5 in our case, a detailed study on different ratios needs to be further investigated.

Figure 2d reveals that N-CNTs grow randomly on the surface of NF covered by N-graphene and Figure 2e shows a detailed single N-CNT chosen from Figure 2d. Also, to further identify the integration between CNTs and graphene, some high-resolution SEM images in different spots of the as-prepared samples are shown in Figure S3a–h (Supplementary) and it is obvious that CNTs and graphene formed a 3D whole by seamless connection in the interface between them. This magnified image of a single N-CNT shown in Figure 2e reveals that the CNT with a diameter of about 20 nm projects vertically above the underlying N-doped graphene surface. Figure 2e also shows some bumps on the N-doped graphene surface, which we ascribe to Ni nanoparticles. Simultaneously, some white dots appear on the tip of N-CNTs, which is investigated by following TEM images to be Ni catalysts. The Ni nanoparticles are derived from NF and produced after the etching process of NH_3_. This corresponds to the “tip growth” mechanism. In this mechanism, with the temperature increasing, carbon atoms continue to diffuse to the surface of metal catalyst. Subsequently, when the carbon atoms are saturated, they precipitate from the bottom of the metal particles with the structure of N-CNTs. As shown in Figure 2f, the N-graphene layers covers the surface of NF and the N-graphene serves as the comparison of N-CNTs/N-graphene in the later characterization. Moreover, the high-resolution SEM image of graphene sheet is shown in Figure S4. The crack of NF, forming during the high temperature reaction, is obviously observed and the N-doped graphene sheets seamlessly coats on the framework of NF, suggesting good contact between graphene sheet and metal substrate. Notably, ripples and wrinkles are formed on the graphene sheets, indicating the features of the 2D structure.

**Figure 3:**
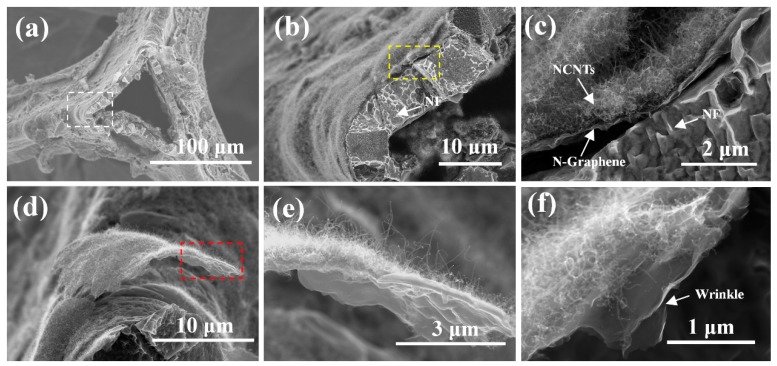
(**a**) SEM image of the top view of a triangle fracture plane of NF; (**b**,**c**) the high magnification of the edge of the fracture plane of NF; (**d**) A flake composed of N-CNTs/N-graphene cocked from the surface of NF; (**e**,**f**) The high magnification SEM images of flake.

To further confirm the whole structure of N-CNTs/N-graphene, we try to observe the material from different view. Figure 3a shows a triangle fracture plane which maybe caused during the preparation process of sample and the surface of fracture plane is covered by thin nanostructure composed of N-CNTs and N-graphene. According to the white box in the Figure 3a, more detailed SEM image is provided in Figure 3b,c. We can observe that the N-CNTs/N-graphene adhere to the surface of NF. In addition, the thickness of thin N-CNTs/N-graphene sheet is about 1 μm and the thickness of NF is around 5 μm. As shown in Figure 3c, the N-CNTs are uniformly and densely grown on the surface of N-graphene layers. Compared to previous work by Zhang et al., where the N-doped CNTs displayed a different diameter distribution and higher degree of entanglement on the surface of graphene sheets [35], our one-step process shows better uniform growth and dispersion of CNTs in the surface of graphene with optimal conditions. Meanwhile, Figure 3d,e provide high and low-magnification of a freestanding flake composed of N-CNTs/N-graphene structure which appears as hair like follicles on the surface of NF. The N-CNTs grown on the surface of N-graphene seamlessly connect with N-graphene. This consequence is consistent to the result from Figure 3b,c. Freestanding and multilayer N-graphene are shown in Figure 3f and ripples and wrinkles are formed on the N-graphene films due to the difference between the thermal expansion coefficients of nickel and graphene [36].

**Figure 4:**
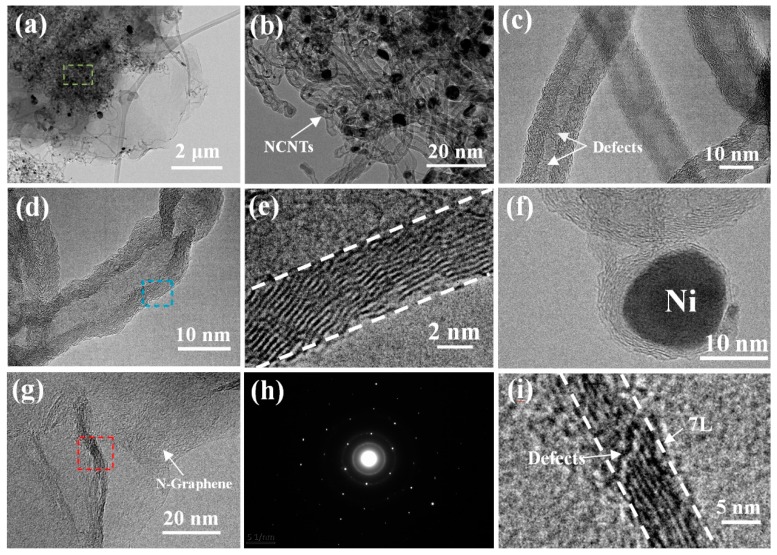
(**a**) TEM image of N-CNTs/N-graphene structure; (**b**) HRTEM images about N-CNTs taken from the box in (**a**); (**c**) TEM image of defects in the N-CNTs; (**d**,**e**) HRTEM of the single multiwall N-CNT; (**f**) The mental catalyst on the tip of N-CNTs; (**g**,**h**) The TEM image and the selected electron diffraction pattern (SAED) of N-graphene, respectively; (**i**) Shown the number of wall of N-graphene in (**g**).

In order to further observe the internal structure of the 3D N-CNTs/N-graphene material, TEM and HRTEM images are provided to confirm the N-CNTs and N-graphene. As shown in the low-magnification TEM image of Figure 4a, there are a lot of N-CNTs randomly compact and stack together above N-graphene which exhibit the typical 2D structure feature. Figure 4b provides the enlarge TEM image of N-CNTs and we can see that Ni catalysts are distributed on the tip of N-CNTs, which is corresponding to the “tip growth” mechanism. Furthermore, from the high-magnification TEM image (Figure 4d) of the N-CNT, it exhibits a typical morphology of multi-walled CNT. The diameter of N-CNTs and Ni catalysts are determined to be 20–25 nm and 15–20 nm (illustrated from Figure 4e,f, respectively.) Interestingly, there are many defects on the walls of N-CNTs as shown in Figure 4c, which results mainly from the successful doping of nitrogen atom into the CNTs [37]. The N-graphene nanosheets are shown in Figure 4g; some wrinkles [38] emerge on the surface of graphene nanosheets, which is due to defective architecture formed during the sample preparation ultrasound process or the doping of nitrogen atoms [19]. The selected area electron diffraction (SAED) in Figure 4h reveals that the N-graphene is highly graphitic. Furthermore, the well-defined diffraction rings and spots are fully indexed to the typical hexagonal lattice of carbon in N-CNTs/N-graphene, demonstrating the well-crystallized structure of N-CNTs/N-graphene prepared via using nickel foam and melamine at 800 °C [39]. According to the red box in Figure 4g, the HRTEM in Figure 4i show the number of layers of N-doped graphene and there are some wrinkles on the walls of N-graphene. The wrinkles are attributed to successful doping of nitrogen originated from melamine and this enables rapid electron transport of graphene. As shown in Figure S5a, the sample do not inherit the porous 3D interconnected structure of NF, however, the 3D structure composed of N-CNTs and N-graphene sheet is restored. The N-doped CNTs still densely and uniformly grow on the surface of N-doped graphene sheet (Figure S5b,c), indicating the strong connection between CNTs and graphene. Moreover, the N-doped graphene sheet serves as the supports of CNTs and Ni nanoparticles, which facilitate the nucleation and growth of CNTs.

**Figure 5:**
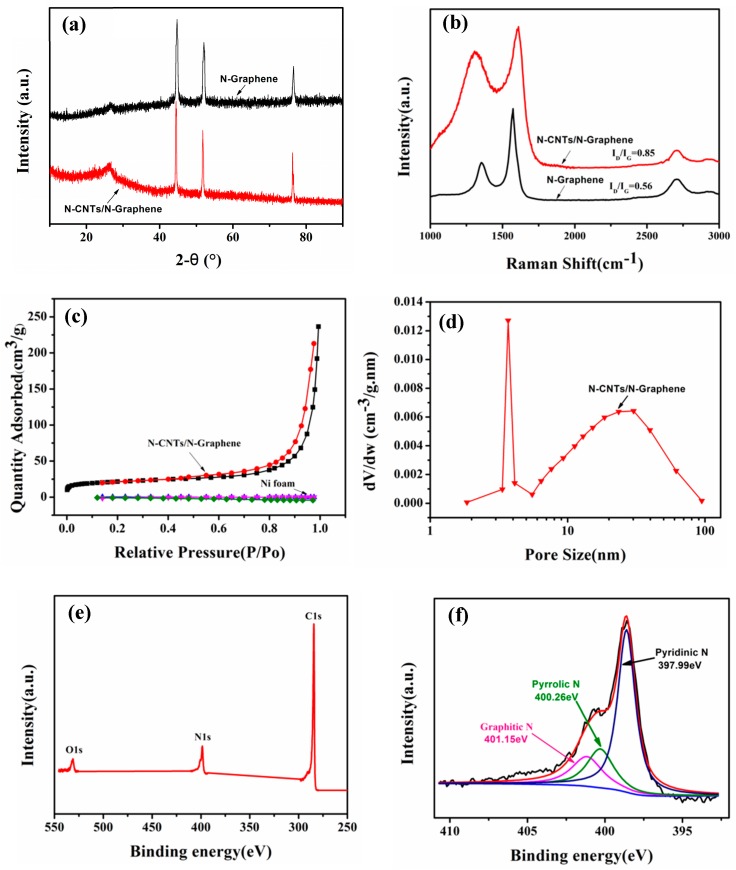
(**a**) XRD of N-CNTs/N-graphene and N-graphene; (**b**) Raman spectra of N-CNTs/N-graphene and N-graphene on the surface of NF; (**c**) N_2_ adsorption-desorption curves of N-CNTs/N-graphene and NF; (**d**) Pore distribution of N-CNTs/N-graphene; (**e**,**f**) XPS spectrum and high-resolution XPS of N1s of N-CNTs/N-graphene.

To further investigate the characteristics of 3D N-CNTs/N-graphene on the surface of NF, we use the X-ray diffraction to demonstrate the elemental analysis and crystalline degree shown in Figure 5a. The characteristic peaks of N-graphene are identified at 26.1°, consistent with the (002) plane of graphite carbon. However, the three strong peaks at 44.5°, 52.5° and 76° are attributed to the presence of Ni [40]. And from the red curve of N-CNTs/N-graphene, four characteristic diffraction peaks can also be observed at 26.1°, 44.5°, 52.5° and 76°, respectively. However, the characteristic peak of N-CNTs/N-graphene at 26.1° is sharper than that of N-graphene, meaning the more graphite amount and well crystalline degree due to the presence of N-CNTs [28]. The result shows that CNTs and N-doped restore the graphitic crystal structure [41]. Figure 5b shows the Raman spectroscopy of N-CNTs/N-graphene and N-graphene, the three distinct peaks located at 1340 cm^−1^, 1580 cm^−1^ and 2700 cm^−1^ respectively are similar to those Raman peaks of N-doped graphene sheets by CVD method previously reported by Qu et al. [42].The peak located at 1340 cm^−1^ corresponds to the D band of graphitic carbon, which is associated with the number of defects in the crystalline structure of the carbon nanotube and graphene layers [43]. The peak located in 1580 cm^−1^ is G peak, which is originated from the E_2g_ vibrational of the sp^2^-bonded carbon atoms. The intensity ratio of I_D_/I_G_ indicate the defects in the graphene structure and degree of graphitization. The Raman spectrum also reveals a weak 2D peak located at 2700 cm^−1^ with a I_2D_/I_G_ of 0.23, confirming the N-doped graphene sheet is multilayer, which is consistent with the result shown in Figure 4i. The intensity ratio (I_2D_/I_G_) of N-CNTs/N-graphene is smaller than that of N-graphene, which is corresponding to the presence of N-CNTs. Moreover, the intensity ratio (I_D_/I_G_) of N-CNTs/N-graphene is 0.85, which is higher than the intensity ratio (I_D_/I_G_ = 0.56) of graphene due to the high-level defects from N-doping. Compared to the N-doped CNTs/graphene hybrids (I_D_/I_G_ = 0.8) fabricated by Yan et al., our 3D hybrids (I_D_/I_G_ = 0.85) include more defects induced by highly nitrogen doping and possess higher atomic ratio of nitrogen [28]. These unique spectrum properties reveal the defective structure of N-CNTs/N-graphene due to nitrogen doping.

The Brunauer-Emment-Teller (BET) specific surface areas of N-CNTs/N-graphene and NF are measured from the nitrogen adsorption and desorption isotherms in Figure 5c. The nitrogen adsorption and desorption isotherms of N-CNTs/N-graphene with a hysteresis loop at a relative pressure P/P_0_ from 0.53 to 0.94 and it belongs to the typical IV curve [44], which usually appears in mesoporous solids. The specific surface area of N-CNTs/N-graphene is 77.4 m^2^/g which is higher than that of the N-doped graphene (the specific surface area is 6 m^2^/g) via thermal annealing graphite oxide with melamine and the N-doped nanotube (the specific surface area is 68 m^2^/g) using melamine as nitrogen source in the previous report, indicating the CNTs within N-CNTs/N-graphene could effectively connect with graphene to integrate the 3D hybrids and enhance dispersion of tubes and graphene sheets [35,41]. The pore volume of N-CNTs/N-graphene is 0.35 cm^3^/g and NF is 0.13 cm^3^/g, the increased pore volume is mainly due to the N-CNTs and N-graphene grown on the surface of NF. Pore size distribution curve is utilized by using Barrett-Joyner-Halenda (BJH) to further identify the mesoporous structure of N-CNTs/N-graphene. Figure 5d further demonstrate the pore sizes derived from the N_2_ desorption peaks mainly spanned from 2 nm to 50 nm but are narrowly distributed around 4nm and 25 nm in the hybrids, which is in good agreement with similar nitrogen-rich carbon nanotube-graphene hybrids in the previous report by Ding et al. [7].

X-ray photoelectron spectroscopy (XPS) is used to investigate the elemental composition and the structure of N-CNTs/N-graphene. C 1s peak located at about 284 eV, N 1s peak at about 399.8 eV and O 1s peak at about 531.3 eV can be obtained on the full peak of X-ray photoelectron spectroscopy (Figure 5e). Remarkably, among the three atomic percentages (C, N, O) in N-CNTs/N-graphene, the nitrogen atom is up to 12.37% (as shown in Table S1). The result, much higher than that of the previous studies (between 0.53% and 10.1% in nitrogen atomic percentage) about N-doped CNTs or graphene by using CVD methods [42,45], is probably attributed to the easier incorporation into carbon matrix of nitrogen from pyrolysis of melamine. Also, the electronic transport and chemical property of sample may be improved due to the high level of N-doping. The bonding configurations of nitrogen atoms are identified by high-resolution N 1s spectra. The analysis shows three peaks at different binding energy. The two peaks located at 397.99 eV and 400.26 eV, corresponding to pyridinic-N and pyrrolic-N, which could contribute to the π-conjugated system with a pair of p-electrons in the layers of carbon nanotube and graphene in the as prepared N-CNTs/N-graphene [41]. Furthermore, the pyridinic-N shows strongest intensity among three styles of doped nitrogen and it is the main component in the as-obtained N-CNTs/N-graphene (as shown in Table S2). The previous studies have suggested the pyridine-like N structures not only are responsible for the metallic behavior and the prominent features near the Fermi level [46] but also beneficial to electronic conductivity and catalytic activity of oxidation-reduction reaction (ORR) by adsorption of oxygen molecules and intermediates [47]. Besides, when the nitrogen atoms substitute carbon atom within the graphene layers in the form of graphitic-N, the peak is shown at 401.15 eV. And the intensity of graphitic-N reveals the successful nitrogen doping process of N-CNTs/N-graphene in the one-step CVD process.

## 4. Conclusions

In summary, we have reported a facile one-step CVD technique approach to fabricate 3D N-CNTs/N-graphene nanomaterial using low-cost industrial melamine as a single carbon and nitrogen source. The N-CNTs/N-graphene are synthesized from one-dimensional N-doped carbon nanotubes and two-dimensional N-doped graphene which grown on the surface of nickel foam possessing interconnected porous 3D structure at 800 °C. The one-dimensional N-doped carbon nanotubes densely distribute on the surface of two-dimensional graphene sheet. The graphene sheet serves as supports of N-doped CNTs and Ni nanoparticles, which lead to nucleation and growth of CNTs and the CNTs in turn prevent the graphene sheets from aggregating. The results also show that defects appear in the wall of CNTs and graphene sheets, which means the successful nitrogen doping process of N-CNTs/N-graphene in the one-step CVD process. Remarkably, the atomic percent of nitrogen is up to 12.7%, which is higher than that of the previous reports on similar materials. And pyridinic-N is the main bonding configurations of nitrogen atoms in the 3D hybrids. The doped nitrogen induces more defects and active sites on the surface of carbon framework but also effectively improves the electronic properties and surface wettability. As a result, the N-CNTs/N-graphene nano-architecture may be suitable for many energy-conversion and energy-storage materials, for example, Li-ion secondary batteries, supercapacitors and Li-S batteries.

## Supplementary Materials

The following are available online at http://www.mdpi.com/2079-4991/8/9/700/s1, Figure S1: (a), (b) and (c) SEM image of N-doped CNTs by using different mass ratio of NF and melamine, Figure S2: SEM image of melamine on NF at 400 °C, Figure S3: (a–h) high-magnification SEM images showing the integration between CNTs and graphene in different spots of samples, Figure S4: High-magnification SEM image of graphene sheet on NF, Figure S5: (a), (b), and (c) Low-magnification and high-magnification SEM images of sample after removing NF, Table S1: Atomic composition of N-CNTs/N-graphene, Table S2: Different bonding configurations of N in N-CNTs/N-graphene.
