# Supplementary material for: One-Step Chemical Vapor Deposition Synthesis of 3D N-doped Carbon Nanotube/N-doped Graphene Hybrid Material on Nickel Foam
Source: Nanomaterials (Basel). 2018 Sep 7;8(9):700. doi: 10.3390/nano8090700 (PMC6164574; doi:10.3390/nano8090700)
Supplement: Supplementary file 1 [file nanomaterials-08-00700-s001.pdf]

Supporting Information

Table S1. Atomic composition of N-CNTs/N-graphene.

Sample	C	N	O
atomic (%)	84.53	12.37	3.09

Table S2. Different bonding configurations of N in N-CNTs/N-graphene.

Sample	Graphitic-N	Pyridinic-N	Pyrrolic-N
(%)	14.3	68.7	16.9

Different mass ratios between NF and melamine for fabricating uniform N-CNTs/N-graphene

To obtain uniform N-CNTs growth in this study, we carried out experiments by using different mass ratio between NF and melamine (1:1, 1:5, 1:10), as displayed in the following Figure S3. When using 1:1, only a small quantity of N-doped CNTs were observed, as indicated in the SEM image of Figure S3a, revealing that low content melamine will produce the sparse N-doped CNTs in the resulting product that probably due to the insufficient carbon source from the pyrolysis of melamine. When the melamine content is increased to 1:5, uniform and dense N-doped CNT growth was fabricated, as shown in Figure S3b. As displayed in Figure S3c, non-uniform N-doped CNTs were observed when the mass ratio NF and melamine is increased to 1:10, which is probably due to the excess carbon content from the pyrolysis of melamine, resulting in the formation of Ni NPs with different size and eventually non-uniform growth of N-doped CNTs.

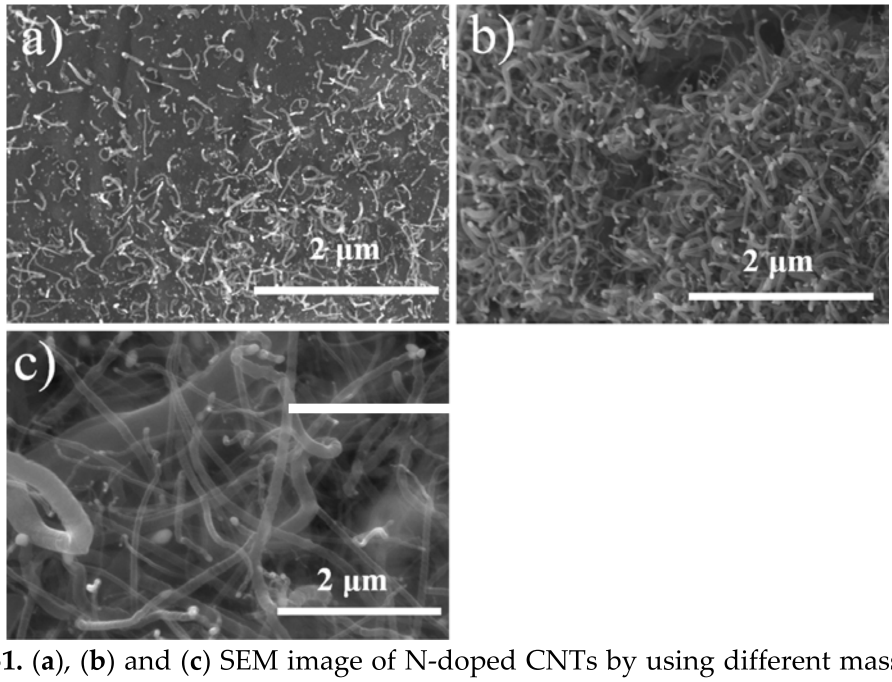

Figure S1. (**a**), (**b**) and (**c**) SEM image of N-doped CNTs by using different mass ratio of NF and melamine.

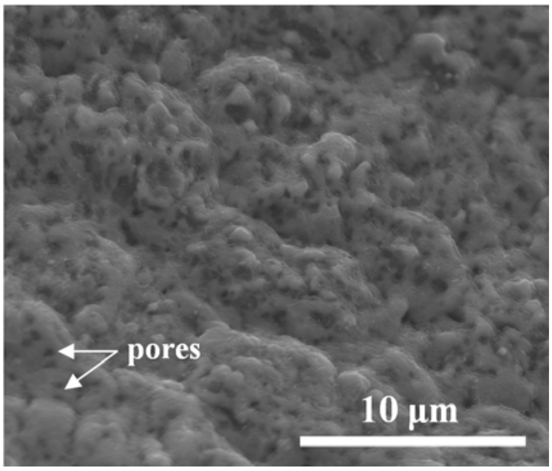

Figure S2. SEM image of melamine on NF at 400 °C.

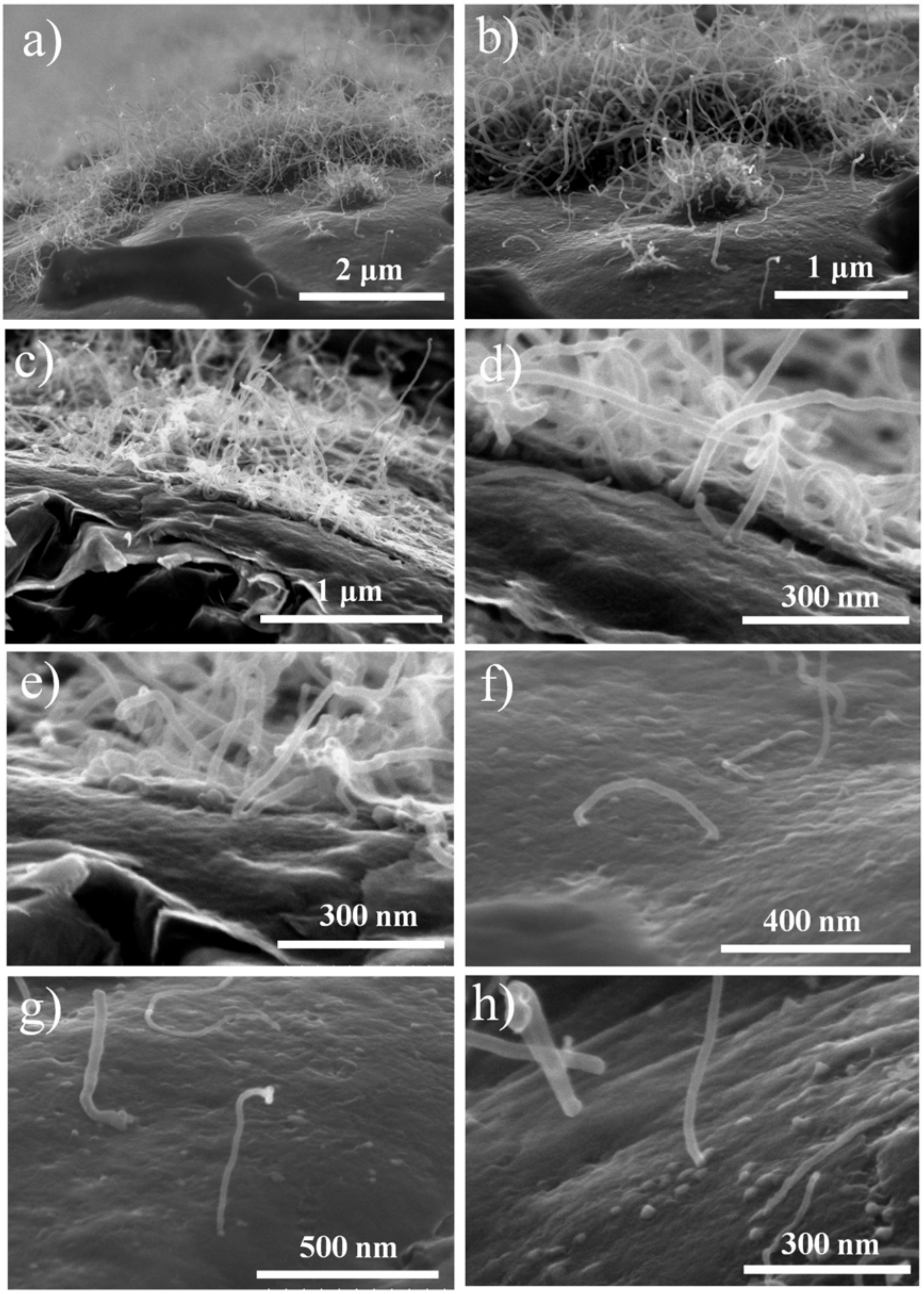

Figure S3. (**a**–**h**) high-magnification SEM images showing the integration between CNTs and graphene in different spots of samples.

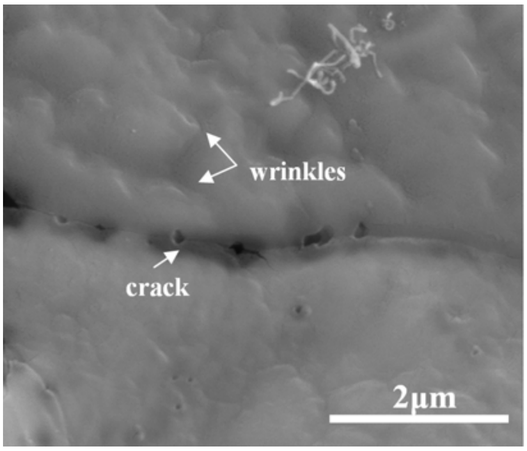

Figure S4. High-magnification SEM image of graphene sheet on NF.

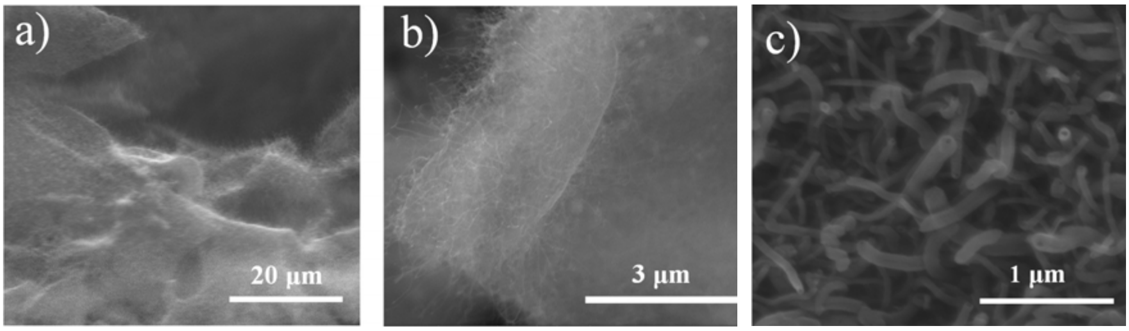

Figure S5. (**a**), (**b**), and (**c**) Low-magnification and high-magnification SEM images of sample after removing NF.
